# Consuming Almonds vs. Isoenergetic Baked Food Does Not Differentially Influence Postprandial Appetite or Neural Reward Responses to Visual Food Stimuli

**DOI:** 10.3390/nu9080807

**Published:** 2017-07-27

**Authors:** R. Drew Sayer, Jaapna Dhillon, Gregory G. Tamer, Marc-Andre Cornier, Ningning Chen, Amy J. Wright, Wayne W. Campbell, Richard D. Mattes

**Affiliations:** 1 Anschutz Health and Wellness Center, University of Colorado—Denver, Anschutz Medical Campus, Aurora, CO 80045, USA; Marc.Cornier@ucdenver.edu; 2 Department of Nutrition Science, Purdue University, West Lafayette, IN 47907, USA; jdhillon5@ucmerced.edu (J.D.); amyjwright2@purdue.edu (A.J.W.); campbellw@purdue.edu (W.W.C.); mattes@purdue.edu (R.D.M.); 3 Department of Molecular Cell Biology, University of California, Merced, CA 95343, USA; 4 Weldon School of Biomedical Engineering, Purdue University, West Lafayette, IN 47907, USA; gtamer@purdue.edu; 5 Division of Endocrinology, Metabolism and Diabetes, Department of Medicine, University of Colorado—Denver, Anschutz Medical Campus, Aurora, CO 80045, USA; 6 Department of Statistics, Purdue University, West Lafayette, IN 47907, USA; Ningning0404@gmail.com

**Keywords:** almonds, fMRI, reward, appetite, obesity

## Abstract

Nuts have high energy and fat contents, but nut intake does not promote weight gain or obesity, which may be partially explained by their proposed high satiety value. The primary aim of this study was to assess the effects of consuming almonds versus a baked food on postprandial appetite and neural responses to visual food stimuli. Twenty-two adults (19 women and 3 men) with a BMI between 25 and 40 kg/m^2^ completed the current study during a 12-week behavioral weight loss intervention. Participants consumed either 28 g of whole, lightly salted roasted almonds or a serving of a baked food with equivalent energy and macronutrient contents in random order on two testing days prior to and at the end of the intervention. Pre- and postprandial appetite ratings and functional magnetic resonance imaging scans were completed on all four testing days. Postprandial hunger, desire to eat, fullness, and neural responses to visual food stimuli were not different following consumption of almonds and the baked food, nor were they influenced by weight loss. These results support energy and macronutrient contents as principal determinants of postprandial appetite and do not support a unique satiety effect of almonds independent of these variables.

## 1. Introduction

Nuts are widely recognized as a potential component of a healthful dietary pattern, but the high energy and fat content of nuts raise continued concerns regarding their role in weight management [1]. These concerns persist despite evidence from epidemiologic studies indicating an inverse relationship between nut consumption and body weight [2,3,4] and meta-analytical findings from clinical trials [5] demonstrating that nut consumption does not increase the risk of weight gain and obesity. Potential mechanisms contributing to the inverse or neutral relationship between nut consumption and risk of obesity and weight gain include increased satiety, lower-than-predicted energy extraction, and possible enhancement of resting energy expenditure [6]. 

As regards almonds specifically, daily intake of 1337 kJ/day (320 kcal) of almonds for six months did not significantly increase body weight [7], and this was supported by a mechanistic metabolic study where daily consumption of 1440 kJ/day (344 kcal) of almonds for 10 weeks did not result in weight change [8]. Several additional clinical trials revealed that almonds may be included in an energy restricted diet without compromising weight reduction [7,9,10,11]. Further, 4- and 12-week trials indicate that consumption of almonds specifically as a snack reduced hunger and desire to eat ratings and did not increase energy intake or body weight [12,13]. Collectively, these data suggest that almonds promote satiety and demonstrate that their daily consumption does not lead to increased body weight or undermine the effectiveness of a weight loss regimen. 

However, many of the studies investigating the impact of acute almond consumption on ingestive behavior have used a negative control study design (addition of almonds vs. no food) [12,14] or have inferred a satiety effect from observed reductions in foods eaten throughout the day when almonds are included in the diet [8]. One study that compared postprandial hunger among eight different preloads (peanuts, peanut butter, almonds, chestnuts, chocolate, rice cakes, pickles, and no load) found that reductions in hunger were not different among energy-equivalent foods and concluded that energy content is the primary determinant of a food’s impact on perceived appetite [15]. Other important mediators of appetite sensations and ingestive behavior include macronutrient distribution (especially protein content) [16,17], fiber content [18], and food form (i.e., liquid vs. solid) [19,20]. Food form may be especially relevant for peanuts and tree nuts that are often processed to form butters (i.e., peanut butter, almond butter) and other food products. For example, daylong fullness was highest when whole almonds were consumed compared to other almond products or the control condition with no almond product consumed [21]. Studies directly comparing the appetitive effects of whole almonds and relative solid foods that are matched for energy, macronutrient, and fiber contents would allow for the investigation of whether almonds demonstrate a unique satiety effect (i.e., aside from observed effects of energy content, macronutrient distribution, fiber content, and food form on appetite) that may partially explain their neutral or beneficial effects on weight management.

Energy intake is driven by an interaction between reportedly homeostatic (i.e., leptin action in the hypothalamus) and non-homeostatic or reward-related neural pathways (i.e., mesocortolimbic dopamine system and associated brain regions [22]). Human functional magnetic resonance imaging (fMRI) studies have consistently demonstrated that visual food stimuli elicit greater responses in reward-related brain structures including the insula, striatum, amygdala, and orbitofrontal cortex of overweight/obese vs. normal weight adults [23,24,25]. Studies in adults with normal weight status suggest that neural responses to visual food stimuli are attenuated in the postprandial state [24] and could represent a more objective measure of motivation/desire for food. However, data regarding postprandial attenuation of neural responses to visual food stimuli in adults with overweight/obesity are mixed [24,26,27] and require further investigation.

Therefore, the primary aim of this study was to investigate the effects of consuming 28 g of almonds vs. an isoenergetic baked food product with a similar macronutrient distribution and fiber content on postprandial appetite ratings (hunger, desire to eat, and fullness) and neural responses to visual food stimuli in brain regions associated with reward and motivated behavior in adults with overweight/obesity. Neural responses to visual food stimuli were measured with fMRI and a priori brain regions of interest included the bilateral insula, amygdala, orbitofrontal cortex, caudate, and putamen [23,24]. We hypothesized that reductions in hunger and desire to eat and increases in fullness would not differ following the consumption of almonds and the isoenergetic baked food, and that postprandial neural responses to visual food stimuli (90 min after ingestion) would not differ following the consumption of these foods. These hypotheses were based on documented effects of energy content [15], macronutrient distribution [16,17], fiber content [18], and food form [19,20,21] on appetite control and ingestive behavior. The design of the current study allowed for the investigation of a potential unique satiety effect of almonds that could not be explained by these well-documented factors.

## 2. Materials and Methods

### 2.1. Subjects

**Figure 1:**
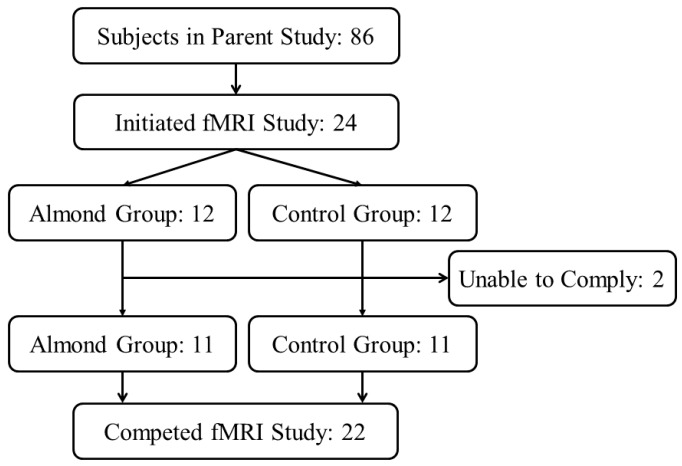
Study recruitment flow diagram.

Adults with overweight/obesity who had provided written informed consent to participate in a 12-week behavioral weight loss intervention (NCT02360787) were given the option to provide additional data for the current study during their concurrent participation in the parent intervention. Twenty-four participants from the parent weight loss intervention opted to participate and provided written informed consent for the current study. All study procedures and documents were approved for use by the Purdue University Biomedical Institutional Review Board. Twenty-two participants (19 female and three male) completed all procedures for the current analysis (Figure 1). One subject each from the almond and control group of the parent intervention (see Section 2.2) were withdrawn from the study for failing to comply with study procedures of the parent intervention. Inclusion criteria for the fMRI study were male or female sex, age 18–60 years, BMI 25.0–40 kg/m^2^, no diabetes, no nut allergies, no claustrophobia, and no implanted ferromagnetic metal or pacemaker/defibrillator. 

### 2.2. Experimental Design of Parent Weight Loss Intervention

Findings from the parent intervention trial were published previously [11]. As part of the parallel-design weight loss intervention, participants were randomly assigned to either consume almonds daily or refrain from eating all nut products for the duration of the study except on testing days described in Section 2.3. All participants received dietary counseling from a registered dietitian to consume an energy-restricted diet. Participants in the almond group were given almonds to consume daily, which provided 15% of energy in their individualized energy-restricted diet. Energy from the almonds was accounted for during dietary prescription and counseling to achieve a 500 kcal/day deficit in both the almond and control groups. No study foods were provided to participants in the control group with the exception of the almonds and baked food that was provided on the 4 testing days for the fMRI study. Equal numbers of participants from the almond (*n* = 12) and control (*n* = 12) groups from the parent weight loss trial were recruited for the fMRI study to achieve balance between the groups, and group assignment was included in statistical models as a covariate (see Section 2.7). However, the study was not statistically powered to determine differences in appetitive or neural responses between the almond and control groups.

### 2.3. Experimental Design of the fMRI Study

Participants completed two testing days prior to beginning the weight loss intervention (pre-intervention) and two testing days during weeks 8–12 of the intervention (post-intervention) in a randomized crossover manner. Testing days were separated by at least seven days (mean: 8.6 days), with the exception of one subject whose post-intervention testing days were three days apart due to schedule conflicts. Participants were given instruction to avoid vigorous exercise for at least 48 h prior to testing days. On all testing days, participants were provided with and asked to consume a standard lunch (25% of estimated daily energy requirement) at 1200 h or 1300 h and to arrive at the Purdue University MRI Facility 5 h after lunch (1700 h or 1800 h). Participants were instructed not to consume any foods or beverages other than water during this 5-h period. Upon arrival, preprandial appetite (hunger, desire to eat, and fullness) and fMRI assessments were completed. Participants then consumed either 28 g of lightly salted, roasted whole almonds or a 40 g baked food product with equivalent energy, macronutrient distribution, and fiber content (Table 1). The recipe for the baked food product was created by the research dietitian (AJW) and resembled a small biscuit. The baked food product was produced in the metabolic research kitchen at Purdue University, which is supported by the NIH through the Indiana Clinical and Translational Sciences Institute. Postprandial appetite assessments were completed immediately after (0 min) and 30, 60, and 90 min after consuming the almonds or baked food. Postprandial fMRI assessments were initiated immediately after completing the 90-min appetite assessment. The alternate food was consumed on the 2nd testing day and this randomized process was repeated for the third and fourth testing days during post-intervention testing. The order of consuming the almonds and baked food product was determined using computerized randomization software [28] both at pre-intervention and post-intervention.

### 2.4. Body Mass and Composition

Body mass and composition were measured at pre-intervention and post-intervention using dual X-ray absorptiometry (General Electric Lunar Prodigy, Version 15.10.035 enCORE iDXA software, Madison, WI, USA).

### 2.5. Appetite Assessments

Appetite (hunger, desire to eat, and fullness) ratings were completed using visual analog scales (VAS) [29] by Adaptive Visual Analog Scales software (Neurobehavioral Laboratory and Clinic, San Antonio, TX, USA) [30]. Participants reported their level of hunger, desire to eat, and fullness, by clicking/marking on a 100 mm scale with end descriptors ranging from “Not at all” to “Extremely”.

### 2.6. fMRI Data Acquisition, Preprocessing, and Analysis

Detailed methods for fMRI data acquisition, preprocessing, and analysis are available elsewhere [27,31]. Neural responses to visual food stimuli were measured using a 3.0 Tesla magnetic resonance scanner (General Electric, Signa HDx, Milwaukee, WI, USA) housed at the Purdue University MRI Facility. Visual stimuli were presented using VisualSystem goggles (NordicNeuroLab, Bergen, Norway) and PsychoPy, Version 1.76.00 software [32]. Visual stimuli were images of food of high hedonic value and neutral nonfood objects, which were validated [33] and used previously [27,34,35,36,37]. 

Three functional runs with three blocks of visual food stimuli and three blocks of visual nonfood stimuli each were performed during each fMRI assessment. Each block of visual stimuli lasted 30 s and included 10 images presented for 2.5 s each with a 0.5 s fade between each image. Blocks of a low-level baseline stimulus (fixation cross) lasting 16 s each were presented before the first visual stimuli block, in between blocks of visual stimuli, and after the final visual stimuli block. Functional images were acquired with an echo-planar gradient-echo T2* blood oxygenation level dependent (BOLD) contrast sequence, with TR = 2000 ms, TE = 30 ms, 642 matrix, 20 cm^2^ field of view, 40 slices to cover the whole brain, 3.1 mm thick, and no gap between slices. A high resolution, T1-weighted anatomical scan was completed after functional imaging for co-registration with functional images.

Preprocessing and first-level data analyses (food vs. nonfood BOLD contrasts) were completed using AFNI [38]. Preprocessing steps included slice-time correction, motion correction, smoothing (4.0 Gaussian blur), and normalization of the signal. Preprocessed functional runs were then aligned with the anatomical scan. Censor files were created to identify volumes within each functional run with excessive head motion (>2.5 mm), which were included in the food vs. nonfood contrast regression model. The resulting food vs. nonfood BOLD contrast was expressed as a β coefficient resulting from the regression model. A priori determined regions of interest were bilateral insula, amygdala, orbitofrontal cortex, caudate, and putamen. Spherical regions of interest with 3 mm radii (123 voxels) were created using data from a previous fMRI study of adults with overweight/obesity [31]. 

### 2.7. Statistical Analysis

All statistical analyses were completed using SAS (Version 9.3, SAS Institute Inc., Cary, NC, USA). Unpaired Student’s *t*-tests (PROC TTEST) were used to test for differences in baseline characteristics between participants assigned to the almond and control groups. On the 1st testing day, the first-level, food vs. nonfood BOLD contrast was quantified in each voxel as a β coefficient from the AFNI regression analysis. Next, a mean β coefficient was calculated by averaging the β coefficients of all voxels (123 voxels) in each region of interest to represent the food vs. nonfood contrast for each entire region of interest. Mean β coefficients were then analyzed using single-sample Student’s *t*-tests (PROC TTEST) to determine if the food vs. nonfood contrast was significantly different from zero. A Bonferroni correction was applied to correct for multiple comparisons among 10 a priori brain regions of interest (α = 0.005) to determine statistical significance of the BOLD contrast as in previous studies by our group [27,31]. Only regions of interest demonstrating a significant food vs. nonfood BOLD contrast in the preprandial state on the 1st testing day were considered for further analyses.

Repeated measures ANOVA (PROC MIXED) was used to assess the effects of food (almonds vs. baked food), intervention time (pre-intervention vs. post-intervention), and their interaction term on total area under the curve (AUC) for postprandial hunger, desire to eat, fullness, palatability ratings, and postprandial neural responses to visual food stimuli. AUCs for hunger, desire to eat, and fullness were calculated using the trapezoidal rule. Group assignment in the weight loss intervention was included as a covariate in the model. As an exploratory analysis, hunger, desire to eat, and fullness ratings were assessed using doubly repeated measures ANOVA (PROC MIXED) to test for effects of time relative to food consumption (preprandial vs. 0 min vs. 30 min vs. 60 min vs. 90 min), food (almond vs. baked food), and intervention time (pre-intervention vs. post-intervention). A Tukey-Kramer adjustment for multiple comparisons was used as needed for post-hoc analyses. Baseline participant characteristics are reported as mean ± SEM and outcome data are presented as LSMEANS ± SE and statistical significance was set at α = 0.05 unless otherwise noted.

## 3. Results

### 3.1. Participant Characteristics

Participants were 35 ± 3 years of age with an average BMI of 30.0 ± 1.0 kg/m^2^ and 39.9 ± 1.6% body fat at baseline. Baseline participant characteristics were not significantly different between participants assigned to the almond and control groups for the parent intervention trial (Table 2). Among all participants, body and fat masses were decreased by 1.8 ± 0.6 kg and 1.3 ± 0.5 kg, respectively after the 12-week intervention, and these changes were not different between participants in the almond and control groups. 

### 3.2. Appetite Ratings

**Figure 2:**
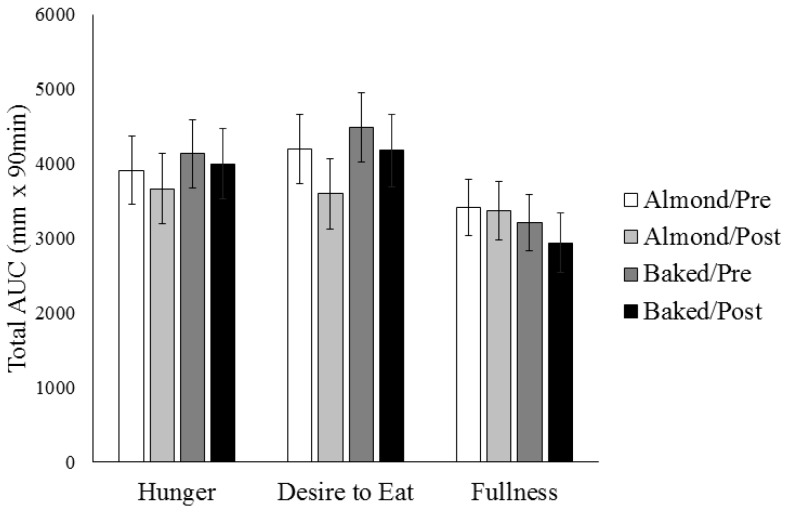
Appetite ratings for almonds and the baked product before and after the 12-week weight loss intervention. Postprandial total AUCs for hunger, desire to eat, and fullness were not different after consuming almonds vs. baked nor at Pre-Intervention vs. Post-Intervention. Abbreviations: AUC, area under the curve.

Preprandial hunger, desire to eat, and fullness ratings were not different on the 4 testing days (data not shown). Postprandial AUCs for hunger, desire to eat, and fullness were not different between almonds and the baked food nor were they influenced by the 12-week weight loss intervention (Figure 2). 

Compared to preprandial ratings (hunger: 47 ± 3 mm, desire to eat: 51 ± 3 mm, fullness: 31 ± 3 mm), hunger (35 ± 3 mm), and desire to eat (38 ± 3 mm) were reduced and fullness increased (42 ± 3 mm) immediately after eating (main effect of time relative to food consumption, *p* < 0.001). Appetite ratings were not differentially influenced following the consumption almonds vs. the baked food product. Appetite ratings 30 min (hunger: 42 ± 3 mm, desire to eat: 45 ± 3 mm, fullness: 34 ± 2 mm), 60 min (hunger: 46 ± 3 mm, desire to eat: 47 ± 3 mm, fullness: 32 ± 2 mm), and 90 min (hunger: 48 ± 3 mm, desire to eat: 50 ± 3 mm, fullness: 30 ± 2 mm) after eating were not different than preprandial ratings. The time courses for hunger, desire to eat, and fullness ratings were not different following consumption of almonds vs. the baked food or at pre-intervention vs. post-intervention. 

### 3.3. Neural Responses to Visual Food Stimuli

**Figure 3:**
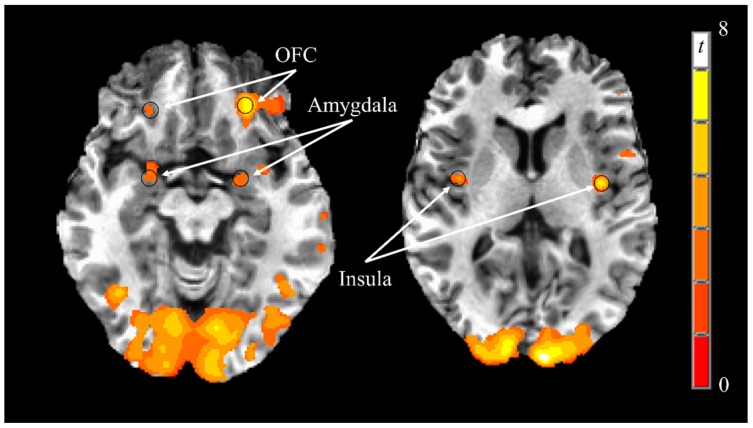
Visual representation of preprandial neural responses to visual food stimuli on Day 1. Greater responses to visual food stimuli vs. nonfood stimuli (PROC TTEST, SAS, Version 9.3; *p* < 0.005) were observed in the bilateral orbitofrontal cortex, amygdala, and insula. Black circles represent functional regions of interest with 3 mm radii within a priori brain regions of interest with known reward functions. Images are in the axial plane and left side of the figure corresponds to the right side of the body and vice versa. Display threshold: *p* < 0.005 and minimum cluster size of 123 voxels. Abbreviations: OFC, orbitofrontal cortex.

On the first testing day, preprandial responses to visual food stimuli were greater than visual nonfood stimuli in the bilateral insula, amygdala, and orbitofrontal cortex (Table 3, Figure 3), but not caudate or putamen. Therefore, postprandial responses and the influence of food consumed (almonds vs. baked food) and time (pre-intervention vs. post-intervention) were only investigated in the insula, amygdala, and orbitofrontal cortex. Postprandial neural responses to visual food stimuli were not different following consumption of either almonds vs. baked foods or when the foods were consumed at pre-intervention vs. post-intervention (Table 4).

## 4. Discussion

The primary aim of the current study was to investigate the impact of almond consumption on postprandial appetitive and neural reward responses to visual food stimuli before and after a 12-week behavioral weight loss intervention. The results of the current study are consistent with our hypotheses of no difference in postprandial appetitive sensations or neural responses to visual food stimuli after consuming almonds and an isoenergetic baked food product.

The satiety value of nuts (including almonds) has been suggested as a potential mechanism by which their consumption does not pose a threat to healthy body weight maintenance [6]. Postprandial hunger and desire to eat ratings were not different following consumption of comparable portions of almonds and a baked food in the current study, which supports previous research demonstrating the importance of energy content and macronutrient distribution for mediating postprandial appetite [15]. However, energy extraction from almonds and other nuts is reduced by their structure and fiber content. In particular, the parenchymal cell walls of nuts must be disrupted by mechanical processing, microbial degradation, and/or enzymatic activity to fully access the energy present in nuts. The fiber present in almonds also may reduce the efficiency of energy extraction by binding fatty acids that are released into the intestinal lumen [39]. While energy extraction and metabolizable energy of the almonds and baked product were not measured in the current study, it is possible that energy extraction may have been reduced in almonds vs. baked food. Therefore, similar postprandial appetite ratings between almonds and the baked food may actually represent a greater suppression of hunger and desire to eat following almond consumption. Future studies should measure fecal energy losses to determine metabolizable energy and energy absorption and match foods on the basis of net energy absorption rather than energy values predicted from Atwater factors to clarify the impact of almond consumption on appetite, ingestive behavior, and/or weight status. 

Interest in the role of the brain’s reward system in the modulation of ingestive behavior and weight status has risen in recent years [24]. Meta-analytical data from fMRI-based studies consistently demonstrate that exposure to visual food cues elicits significant activation in regions of the brain associated with reward and/or motivated behavior including the insula, amygdala, orbitofrontal cortex, striatum, and others. Furthermore, individuals with obesity have greater responses in these regions compared to their lean counterparts [24]. There is limited evidence that meal composition—specifically protein content—may influence postprandial neural reward responses to visual food stimuli. In a study of adolescent breakfast-skipping girls, consumption of a high-protein breakfast reduced responses to visual food stimuli in the insula and pre-frontal cortex compared to a normal protein breakfast [26]. However, high vs. normal protein and fiber intakes at breakfast did not influence postprandial responses to visual food stimuli in a group of overweight young adults [27]. Similar to appetite ratings, postprandial neural responses to visual food stimuli in the insula, amygdala, and orbitofrontal cortex did not differ following consumption of almonds or the baked food in the current study. Collectively, these data suggest that consumption of almonds and a baked food with a similar macronutrient distribution and fiber content did not differentially influence postprandial appetitive drive. 

Strengths of the current study include a high retention rate (22 of 24 participants completed the study) and a total sample size that is consistent with previous fMRI-based studies (*n* = 23 for weight loss interventions in a systematic review by Pursey et al. [24]). Our sample size is also congruent with published recommendations for evaluating the effects of foods and food ingredients on appetite control [40]. The comparison of almonds to a food product of similar estimated energy, macronutrient distribution, and fiber content allowed us to investigate whether the reportedly high satiety value of almonds could be explained by these properties. 

While the overall number of participants was appropriate for an fMRI-based study, the relatively small number of participants in the almond and control groups (*n* = 11 each) that completed the study limited our ability evaluate the impact of daily almond consumption on appetite and neural responses to visual food stimuli. Also, our results suggest that 28 g of almonds and the isoenergetic portion of the baked food product (174 kcal each) may not have been sufficient to elicit strong satiety responses, which may have resulted in limited ability to detect differences between the foods. Past studies using larger portions of almonds (i.e., ≥42 g) have shown greater reductions in hunger and/or increases in fullness [12,14,21] and did not cause weight gain when added to the diet [7,8] or negatively influence weight loss during energy restriction [11]. We elected to use 28 g or 1 oz of almonds in the current study because this is the recommended daily intake of nuts in the 2015–2020 Dietary Guidelines for Americans and constitutes a substantial increase in average nut consumption in the United States [1]. We believe that this level of intake was a reasonable representation of what could be achieved under free-living conditions and therefore has the greatest translational potential to impact public health messaging. Other limitations of the current study include a predominance of women in our sample that limits the generalizability of our results. Female participants were not asked to provide information regarding their menstrual cycles, which could alter appetitive sensations [41] and brain responses [42]. 

## 5. Conclusions

In conclusion, consumption of almonds and a portion of a baked food with a similar macronutrient distribution and fiber content resulted in similar postprandial appetite ratings and neural reward responses to visual food stimuli. This held prior to and following 12 weeks of daily almond consumption during weight loss. These results provide further support for the energy content, macronutrient, and fiber contents of a food as the primary drivers of postprandial appetitive sensations, but potential differences in energy extraction should be considered in future studies.

## Figures and Tables

**Table 1:** Nutrient comparison of almonds and baked food product.

Nutrient	Almonds	Bake Food Product
Total Energy (kcal)	174	174
Total Mass (g)	28	40
Energy Density (kcal/g)	6.2	4.4
Carbohydrates (g) ^1^	6	6
Protein (g)	6	5
Fat (g)	14	14
Fiber (g)	3	3

^1^ Quantities of carbohydrates, protein, fat, and fiber are rounded to the nearest gram. Abbreviations: kcal, kilocalories.

**Table 2:** Baseline subject characteristics ^1^.

Variable	Almond Group (*n* = 11)	Control Group (*n* = 11)	Combined (*n* = 22)	*p* Value for Difference ^2^
Female/Male	9/2	10/1	19/3	-
Age (years)	33 ± 4	37 ± 4	35 ± 3	0.48
Body Mass (kg)	82.8 ± 4.1	80.8 ± 4.1	81.8 ± 2.8	0.73
BMI (kg/m^2^)	29.9 ± 1.2	30.0 ± 1.6	30.0 ± 1.0	0.96
% Body Fat	40.8 ± 25.8	38.9 ± 2.2	39.9 ± 1.6	0.55

^1^ Values presented as mean ± SEM. ^2^ Unpaired Student’s *t*-tests (SAS, Version 9.3, PROC TTEST) were used to test for differences in baseline subject characteristics between participants randomized to the almond and control groups. No differences in baseline characteristics were noted between the groups. Abbreviations: BMI, body mass index.

**Table 3:** A priori brain regions of interest demonstrating a significant food vs. nonfood contrast ^1^.

Brain Region	X^2^	Y	Z	*t* Statistic	*p* Value
Insula (L)	−38	−7	6	6.04	<0.0001
Insula (R)	39	−4	4	4.47	0.0002
Amygdala (L)	−23	0	−17	3.26	0.0036
Amygdala (R)	24	0	−18	3.66	0.0014
Orbitofrontal Cortex (L)	−25	35	−18	4.18	0.0004
Orbitofrontal Cortex (R)	23	33	−20	3.77	0.0010

^1^ Mean β coefficients were analyzed by single-sample Student’s *t*-tests (SAS, Version 9.3, PROC TTEST) to determine if the food vs. nonfood contrast was significantly different from 0. A Bonferroni correction was utilized to control for multiple comparisons among 10 a priori brain regions of interest (α = 0.005). The *t* statistics and *p* values presented in the table are reflective of the results of these tests. ^2^ Montreal Neurological Institute Coordinates. Abbreviations: L, left; R, right.

**Table 4:** Intervention effects on postprandial neural responses to visual food stimuli ^1^.

Brain Region	Food	Time	Food × Time
Insula (L)	0.45	0.11	0.41
Insula (R)	0.72	0.08	0.08
Amygdala (L)	0.90	0.49	0.46
Amygdala (R)	0.89	0.31	0.29
Orbitofrontal Cortex (L)	0.81	0.12	0.83
Orbitofrontal Cortex (R)	0.36	0.08	0.33

^1^ Values in table are the resultant *p* values for main effect of food (almond vs. baked food), food **×** time (pre-intervention vs. post-intervention) interaction, and food × time × group (almond vs. control) interaction on postprandial neural responses to visual food stimuli (SAS, Version 9.3, PROC MIXED).

## References

[1] (2015). Dietary Guidelines for Americans 2015–2020.

[2] Bes-Rastrollo M., Wedick N.M., Martinez-Gonzalez M.A., Li T.Y., Sampson L., Hu F.B. (2009). Prospective study of nut consumption, long-term weight change, and obesity risk in women. Am. J. Clin. Nutr..

[3] Bes-Rastrollo M., Sabaté J., Gómez-Gracia E., Alonso A., Martínez J.A., Martínez-González M.A. (2007). Nut consumption and weight gain in a Mediterranean cohort: The SUN study. Obes. Silver Spring Md..

[4] Casas-Agustench P., Bulló M., Ros E., Basora J., Salas-Salvadó J., Nureta-PREDIMED investigators (2011). Cross-sectional association of nut intake with adiposity in a Mediterranean population. Nutr. Metab. Cardiovasc. Dis. NMCD.

[5] Flores-Mateo G., Rojas-Rueda D., Basora J., Ros E., Salas-Salvado J. (2013). Nut intake and adiposity: Meta-analysis of clinical trials. Am. J. Clin. Nutr..

[6] Mattes R.D., Dreher M.L. (2010). Nuts and healthy body weight maintenance mechanisms. Asia Pac. J. Clin. Nutr..

[7] Fraser G.E., Bennett H.W., Jaceldo K.B., Sabaté J. (2002). Effect on body weight of a free 76 Kilojoule (320 calorie) daily supplement of almonds for six months. J. Am. Coll. Nutr..

[8] Hollis J., Mattes R. (2007). Effect of chronic consumption of almonds on body weight in healthy humans. Br. J. Nutr..

[9] Foster G.D., Shantz K.L., Vander Veur S.S., Oliver T.L., Lent M.R., Virus A., Szapary P.O., Rader D.J., Zemel B.S., Gilden-Tsai A. (2012). A randomized trial of the effects of an almond-enriched, hypocaloric diet in the treatment of obesity. Am. J. Clin. Nutr..

[10] Wien M.A., Sabaté J.M., Iklé D.N., Cole S.E., Kandeel F.R. (2003). Almonds vs. complex carbohydrates in a weight reduction program. Int. J. Obes. Relat. Metab. Disord. J. Int. Assoc. Study Obes..

[11] Dhillon J., Tan S.-Y., Mattes R.D. (2016). Almond Consumption during Energy Restriction Lowers Truncal Fat and Blood Pressure in Compliant Overweight or Obese Adults. J. Nutr..

[12] Tan S.Y., Mattes R.D. (2013). Appetitive, dietary and health effects of almonds consumed with meals or as snacks: A randomized, controlled trial. Eur. J. Clin. Nutr..

[13] Zaveri S., Drummond S. (2009). The effect of including a conventional snack (cereal bar) and a nonconventional snack (almonds) on hunger, eating frequency, dietary intake and body weight. J. Hum. Nutr. Diet. Off. J. Br. Diet. Assoc..

[14] Hull S., Re R., Chambers L., Echaniz A., Wickham M.S.J. (2015). A mid-morning snack of almonds generates satiety and appropriate adjustment of subsequent food intake in healthy women. Eur. J. Nutr..

[15] Kirkmeyer S.V., Mattes R.D. (2000). Effects of food attributes on hunger and food intake. Int. J. Obes. Relat. Metab. Disord. J. Int. Assoc. Study Obes..

[16] Halton T.L., Hu F.B. (2004). The effects of high protein diets on thermogenesis, satiety and weight loss: A critical review. J. Am. Coll. Nutr..

[17] Leidy H.J., Clifton P.M., Astrup A., Wycherley T.P., Westerterp-Plantenga M.S., Luscombe-Marsh N.D., Woods S.C., Mattes R.D. (2015). The role of protein in weight loss and maintenance. Am. J. Clin. Nutr..

[18] Wanders A.J., van den Borne J.J., de Graaf C., Hulshof T., Jonathan M.C., Kristensen M., Mars M., Schols H.A., Feskens E.J. (2011). Effects of dietary fibre on subjective appetite, energy intake and body weight: A systematic review of randomized controlled trials. Obes. Rev..

[19] Tieken S.M., Leidy H.J., Stull A.J., Mattes R.D., Schuster R.A., Campbell W.W. (2007). Effects of solid versus liquid meal-replacement products of similar energy content on hunger, satiety, and appetite-regulating hormones in older adults. Horm. Metab. Res. Horm. Stoffwechselforschung Horm. Metab..

[20] DiMeglio D.P., Mattes R.D. (2000). Liquid versus solid carbohydrate: Effects on food intake and body weight. Int. J. Obes. Relat. Metab. Disord. J. Int. Assoc. Study Obes..

[21] Mori A.M., Considine R.V., Mattes R.D. (2011). Acute and second-meal effects of almond form in impaired glucose tolerant adults: A randomized crossover trial. Nutr. Metab..

[22] Berthoud H.R. (2006). Homeostatic and non-homeostatic pathways involved in the control of food intake and energy balance. Obes. Silver Spring Md..

[23] Tang D.W., Fellows L.K., Small D.M., Dagher A. (2012). Food and drug cues activate similar brain regions: A meta-analysis of functional MRI studies. Physiol. Behav..

[24] Pursey K.M., Stanwell P., Callister R.J., Brain K., Collins C.E., Burrows T.L. (2014). Neural responses to visual food cues according to weight status: A systematic review of functional magnetic resonance imaging studies. Front. Nutr..

[25] Burger K.S., Berner L.A. (2014). A functional neuroimaging review of obesity, appetitive hormones and ingestive behavior. Physiol. Behav..

[26] Leidy H.J., Lepping R.J., Savage C.R., Harris C.T. (2011). Neural responses to visual food stimuli after a normal vs. higher protein breakfast in breakfast-skipping teens: A pilot fMRI study. Obes. Silver Spring Md..

[27] Sayer R.D., Amankwaah A.F., Tamer G.G., Chen N., Wright A.J., Tregellas J.R., Cornier M.-A., Kareken D.A., Talavage T.M., McCrory M.A. (2016). Effects of Dietary Protein and Fiber at Breakfast on Appetite, ad Libitum Energy Intake at Lunch, and Neural Responses to Visual Food Stimuli in Overweight Adults. Nutrients.

[28] Random.org. http://www.random.org.

[29] Stubbs R.J., Hughes D.A., Johnstone A.M., Rowley E., Reid C., Elia M., Stratton R., Delargy H., King N., Blundell J.E. (2000). The use of visual analogue scales to assess motivation to eat in human subjects: A review of their reliability and validity with an evaluation of new hand-held computerized systems for temporal tracking of appetite ratings. Br. J. Nutr..

[30] Marsh-Richard D.M., Hatzis E.S., Mathias C.W., Venditti N., Dougherty D.M. (2009). Adaptive Visual Analog Scales (AVAS): A modifiable software program for the creation, administration, and scoring of visual analog scales. Behav. Res. Methods.

[31] Drew Sayer R., Tamer G.G., Chen N., Tregellas J.R., Cornier M.-A., Kareken D.A., Talavage T.M., McCrory M.A., Campbell W.W. (2016). Reproducibility assessment of brain responses to visual food stimuli in adults with overweight and obesity. Obes. Silver Spring Md..

[32] Peirce J.W. (2008). Generating Stimuli for Neuroscience Using PsychoPy. Front. Neuroinform..

[33] Burger K.S., Cornier M.A., Ingebrigtsen J., Johnson S.L. (2011). Assessing food appeal and desire to eat: The effects of portion size & energy density. Int. J. Behav. Nutr. Phys. Act..

[34] Cornier M.A. (2009). The effects of overfeeding and propensity to weight gain on the neuronal responses to visual food cues. Physiol. Behav..

[35] Cornier M.A., Melanson E.L., Salzberg A.K., Bechtell J.L., Tregellas J.R. (2012). The effects of exercise on the neuronal response to food cues. Physiol. Behav..

[36] Cornier M.A., Von Kaenel S.S., Bessesen D.H., Tregellas J.R. (2007). Effects of overfeeding on the neuronal response to visual food cues. Am. J. Clin. Nutr..

[37] Cornier M.A., Salzberg A.K., Endly D.C., Bessesen D.H., Rojas D.C., Tregellas J.R. (2009). The effects of overfeeding on the neuronal response to visual food cues in thin and reduced-obese individuals. PLoS ONE.

[38] Cox R.W. (1996). AFNI: Software for analysis and visualization of functional magnetic resonance neuroimages. Comput. Biomed. Res..

[39] Tan S.Y., Dhillon J., Mattes R.D. (2014). A review of the effects of nuts on appetite, food intake, metabolism, and body weight. Am. J. Clin. Nutr..

[40] Blundell J., de Graaf C., Hulshof T., Jebb S., Livingstone B., Lluch A., Mela D., Salah S., Schuring E., van der Knaap H. (2010). Appetite control: Methodological aspects of the evaluation of foods. Obes. Rev..

[41] Bryant M., Truesdale K.P., Dye L. (2006). Modest changes in dietary intake across the menstrual cycle: Implications for food intake research. Br. J. Nutr..

[42] Van Vugt D.A. (2010). Brain imaging studies of appetite in the context of obesity and the menstrual cycle. Hum. Reprod. Update.

